# Divergent mechanisms underlie Smad4-mediated positive regulation of the three genes encoding the basement membrane component laminin-332 (laminin-5)

**DOI:** 10.1186/1471-2407-8-215

**Published:** 2008-07-29

**Authors:** Dirk Zboralski, Miriam Böckmann, Marc Zapatka, Sabine Hoppe, Anna Schöneck, Stephan A Hahn, Wolff Schmiegel, Irmgard Schwarte-Waldhoff

**Affiliations:** 1 Department of Internal Medicine, Knappschaftskrankenhaus, IMBL, Ruhr-University of Bochum, Bochum, Germany; 2 Department of Internal Medicine, Molecular Oncology, Ruhr-University of Bochum, Bochum, Germany; 3 Department of Gastroenterology and Hepatology, Kliniken Bergmannsheil, Ruhr-University of Bochum, Bochum, Germany; 4 Department of Theoretical Bioinformatics, DKFZ, Heidelberg, Germany

## Abstract

**Background:**

Functional inactivation of the tumor suppressor Smad4 in colorectal and pancreatic carcinogenesis occurs coincident with the transition to invasive growth. Breaking the basement membrane (BM) barrier, a prerequisite for invasive growth, can be due to tumor induced proteolytic tissue remodeling or to reduced synthesis of BM molecules by incipient tumor cells. Laminin-332 (laminin-5), a heterotrimeric BM component composed of α3-, β3- and γ2-chains, has recently been identified as a target structure of Smad4 and represents the first example for expression control of an essential BM component by a tumor and invasion suppressor. Biochemically Smad4 is a transmitter of signals of the TGFβ superfamily of cytokines. We have reported previously, that Smad4 functions as a positive transcriptional regulator of constitutive and of TGFβ-induced transcription of all three genes encoding Laminin-332, LAMA3, LAMB3 and LAMC2.

**Methods:**

Promoter-reporter constructs harboring 4 kb upstream regions, each of the three genes encoding Laminin-322 as well as deletion and mutations constructs were established. Promoter activities and TGFβ induction were assayed through transient transfections in Smad4-negative human cancer cells and their stable Smad4-positive derivatives. Functionally relevant binding sites were subsequently confirmed through chromatin immunoprecipitation.

**Results:**

Herein, we report that Smad4 mediates transcriptional regulation through three different mechanisms, namely through Smad4 binding to a functional SBE site exclusively in the LAMA3 promoter, Smad4 binding to AP1 (and Sp1) sites presumably via interaction with AP1 family components and lastly a Smad4 impact on transcription of AP1 factors. Whereas Smad4 is essential for positive regulation of all three genes, the molecular mechanisms are significantly divergent between the LAMA3 promoter as compared to the LAMB3 and LAMC2 promoters.

**Conclusion:**

We hypothesize that this divergence in modular regulation of the three promoters may lay the ground for uncoupled regulation of Laminin-332 in Smad4-deficient tumor cells in response to stromally expressed cytokines acting on budding tumor cells.

## Background

Functional inactivation of the tumor suppressor Smad4 in colorectal and pancreatic carcinogenesis occurs coincident with the transition of premalignant precursor lesions – adenomas and pancreatic intraepithelial neoplasias (PanINs), respectively, to invasive and metastatic growth [1-4]. The hallmark of invasive growth is loss of the basement membrane (BM) barrier. BMs are specialized sheet-like structures of the extracellular matrix that separate epithelia from the underlying mesenchyme. They are built through interaction of epithelial and mesenchymal cells, which both provide components, mainly the laminins and collagen IV [5]. The epithelial-derived laminins constitute a family of at least 15 different isoforms in mammals, each an α_x_β_y_γ_z _heterotrimer derived from a combination of one, each, out of five α-, three β- and three γ-glycoprotein subunits [6-8]. In the gastrointestinal tract a single-layered epithelial sheet is separated from the underlying mesenchyme through a BM containing laminins- 111, -211, -332, -511 and -521 (laminins 1, 2, 5, 10 and 11), which are expressed in a characteristic regional and development dependent pattern [5,9,10]. Also, in the normal adult pancreas, a single layer of ductal cells – the presumptive precursors of pancreatic adenocarcinomas, is separated from mesenchymal cells through a BM containing laminin-332 (LM-332) [11-13].

Loss of the BM barrier upon the transition to invasive growth can be due either to proteolytic degradation or to decreased synthesis of BM components [14]. Proteolytic degradation of BM in carcinomas has been intensively investigated. It appears to be predominantly executed through proteases expressed mainly by stromal cell types like activated fibroblasts and inflammatory cells, which can be recruited through signals originating from the tumor cells. Molecular mechanisms underlying decreased synthesis of BM components, on the other hand, have rarely been addressed.

To unravel the mechanisms that underlie Smad4-mediated tumor suppression we have established derivatives of Smad4-deficient human colorectal and pancreatic carcinoma cells, in which Smad4 is stably restored through gene transfer. Using this approach we could proof Smad4's tumor suppressor function and could identify Smad4 target genes, among them VEGF and E-cadherin [15-18]. Recently, we have unraveled, that LM-332, composed of α3-, β3- and γ2-chains, is another relevant target structure of Smad4. We have shown that all three genes encoding LM-332, LAMA3, LAMB3 and LAMC2, are under positive transcriptional control of Smad4. Smad4 increased basal and/or TGFβ-induced expression of LM-332 in Smad4-reexpressing colon and pancreatic cancer cells leading to a huge increase in the extracellular release of the heterotrimer and to the deposition in BM-like structures at contact sites with fibroblasts [19].

LM-332 expression is tightly controlled in normal epithelia; adenomas consistently retain normal staining patterns for LM-332 in BMs [20]. In colorectal carcinomas, in contrast, laminin deposition in BM structures becomes discontinuous or is absent suggesting that shut-down of laminin expression is associated with genetic alterations that mediate the transition to invasive growth [8,14,21]. Thus, our finding that the tumor and invasion suppressor Smad4 can act as a positive regulator of LM-332 is consistent with current knowledge.

Here, we wished to further decipher the molecular mechanisms of how Smad4 acts as a positive transcriptional regulator of constitutive and of TGFβ-induced expression of the three genes encoding LM-332. Smad4 encodes an intracellular messenger common to all signaling cascades induced by members of the TGFβ superfamily of cytokines through the canonical pathway [22,23]. Cellular signaling from the TGFβ family is initiated by binding of the ligand to transmembrane receptor serine/threonine kinases, TβRI and TβRII. Activated TGFβ receptors stimulate the phosphorylation of receptor-regulated Smad (R-Smad) proteins, which in turn form complexes with Smad4 that accumulate in the nucleus. Here, the Smad complex can bind to DNA directly, at so-called SBE sites (Smad binding element), but with low binding affinity, only, and can also bind to and interact with a plethora of other transcription factors, coactivators or corepressors among them transcription factors of the AP1 and Sp1 families. High affinity binding of the Smad complex to a promoter is thought to occur through the incorporation of an additional transcription factor into the R-Smad-Smad4 complex, which binds to its respective cognate sequence [22,23]. Adding to the complexity of cellular signaling networks TGFβ besides the canonical pathway fuels into further signaling cascades like the MAP kinase pathway [24,25]. TGFβ has previously been identified as a positive regulator of LM-332 in diverse cell types among them epidermal keratinocytes and gastric adenocarcinoma cells [26,27]. AP1 sites were found to confer TGFβ responsiveness of the LAMA3 and the LAMC2 promoter -encoding for α3 and γ2-chains- in murine keratinocytes and human colon carcinoma cells, respectively [28-31]. No reports, to our knowledge, have yet been published addressing molecular mechanisms of LAMB3 -encoding for β3-chain- promoter regulation.

We have reported earlier, that an SBE site is functional and is involved in Smad4-mediated TGFβ induction of LAMA3 expression [19]. *In silico *analyses detected putative SBE sites also in the promoter regions of the LAMB3 and the LAMC2 genes but have not yet been functionally analyzed.

Here, we present detailed studies of the three promoters of the genes encoding LM-332 in human colorectal adenoma cells and in Smad4-deficient and Smad4-reexpressing colorectal and pancreatic carcinoma cells. We confirm that the SBE site at -1.5 kb confers one part of Smad4-dependent TGFβ induction of LAMA3 expression and that the downstream AP1 sites are additionally involved. On the other hand, whereas each, three, putative SBE sites were identified in the LAMB3 and the LAMC2 promoter through *in silico *analyses, each of these sites proved non-functional. Rather, TGFβ induction is conferred through AP1-sites and through a single Sp1 site in both of these promoters. In summary, our results show, that whereas Smad4 functions as a positive transcriptional regulator of all three genes encoding LM-332, the underlying mechanisms are surprisingly complex and substantially diverse.

## Methods

### Cell culture

The human colorectal carcinoma cell line SW480 and the human pancreatic carcinoma cell line BxPC3 cells were obtained from the American Type Culture Collection. The human colon adenoma cell line LT97-2 was kindly provided by M Marian (Vienna, Austria). LT97 cells were maintained in Ham's F12 medium with supplements as described [32]. All other cells were maintained in Dulbecco's Modified Eagle Medium (DMEM) supplemented with 10% fetal calf serum, 2 mM glutamine, 100 U/mL penicillin and 100 μg/mL streptomycin.

### Smad4 reconstitution and Western blot analysis

The full-length coding sequence of Smad4 was cloned into the pBK-CMV expression vector (Stratagene) as previously described [17] and Smad4 re-expressing SW480 cell clones and negative control transfectants were established by retroviral transduction.

Expression of the Smad4 protein product was analyzed by Western blotting of lysates. Cells were lysed in NP-40 lysis buffer (25 mM Tris HCl, pH 7.4, 0.5% NP-40, 100 mM NaCl, 1 mM EDTA) containing a protease inhibitor cocktail (Roche) and 1 mM PMSF. Proteins were resolved by SDS PAGE and transferred to Immobilon membranes (Millipore). The blots were incubated with monoclonal antibodies against Smad4 (anti-Smad4 B8; dilution 1:500, Santa Cruz), washed with PBS containing 0.05% Tween 20 and incubated with a secondary antibody which was coupled with the fluorescent dye Alexa Flour 680. Signals were detected using the Odyssey Infrared Imaging System (LI-COR Biosciences).

### Vector construction and transient transfection

Promoter fragments upstream of exon1 of the human LAMA3A; LAMB3 and LAMC2 genes were amplified by genomic PCR with primers listed in additional file 1 and cloned into the pGL3-basic vector (Promega). All constructs were sequence-verified. The 0.8 kb deletion construct of the LAMC2-promoter was generated by double-digestion of the LAMA3-2 kb construct with NheI (within theMCS of pGL3 basic) and EcoRI. Point mutations were introduced using the Quick-Change Site directed mutagenesis kit (Stratagene) with the primer oligonucleotides listed in additional file 1. DNA used for transient transfections was prepared with a plasmid Midi Kit (Qiagen, endotoxin-free).

For transient transfections, cells were grown to a confluency of approximately 50–70% in 24-well plates and transfected with 200/400 ng of the respective promoter construct (pGL3-basic, Promega) and 2/4 ng internal control plasmid (phRL-SV40, Promega) using Effectene (Qiagen) in accordance with the manufacturer's recommendations. TGFβ was added when indicated 5 h after transfection at a final concentration of 5 ng/mL. The cells were harvested after 24 h and the luciferase assays were carried out as triplicates using a luminometer (GloMax™ 96 Microplate, Promgea) and the Dual-Luciferase-Reporter Assay System (Promega).

### Chromatin immunoprecipitation

ChIP assays were performed as described in Zapatka et al. [19] with minor modifications. BxPC3 cells were grown to confluence in 150 mm culture dishes and treated with recombinant TGFβ1 (R&D Systems) at a concentration of 5 ng/mL for 90 min. Proteins and DNA were crosslinked by incubating the cells in 1% (v/v) formaldehyde at room temperature for 10 min and the reaction was stopped by the addition of glycine to a final concentration of 0.125 M. Cells were washed twice with Tris-buffered saline (20 mM Tris, pH 7.4, 150 mM NaCl) and lysed in SDS buffer (50 mM Tris, pH 8.1, 0.5% (v/v) SDS, 100 mM NaCl, 5 mM ethylenediaminetetraacetic acid (EDTA), protease inhibitors, Roche). Chromatin was pelleted by centrifugation and resuspended in 0.5 mL of immunoprecipitation buffer (two parts SDS buffer, one part Triton dilution buffer (100 mM Tris, pH 8.6, 100 mM NaCl, 5% (v/v) Triton X-100, 5 mM EDTA) and protease inhibitors (Roche). Chromatin was sonicated (four times for 20 s, using a Bandelin sonoplus sonicator (Bandelin electronic GmbH&Co KG, Germany) to yield genomic DNA fragments with a bulk size of 300–500 bp. Immunoprecipitaton was performed overnight at 4°C with polyclonal antibodies specific for Smad4 (3 μg H-552 and C-20, Santa Cruz). Immune complexes were recovered by adding 50 μL magneto-beads (Dynal, Invitrogen) and incubated for 2 h at 4°C. Then beads were washed successively as described previously [19]. Washed precipitates were incubated overnight at 65°C in elution buffer (TE, 1% SDS, 0.1 M NaHCO3) to reverse the formaldehyde crosslinking. DNA fragments were purified with a QiaQuick Spin Kit (Qiagen) according to the manufacturer's recommendations except that the samples were first mixed with agitation for 30 min with PB buffer. The promoter regions were amplified from 2 μL of the extracted DNA per reaction and using primers listed in additional file 1 in 32 cycles of amplification to yield fragments of ~200 bp length.

### Northern blot analysis

RNA was isolated with a commercial kit (Qiagen). Northern blots and hybridizations were performed as described previously [17]. AP1 probes were prepared by RT-PCR from cell line RNA, using primers listed in additional file 1. For loading control blots were stripped and reprobed for GAPDH. Signal intensities were quantified by PhosphorImage analysis (Packard).

## Results

### Stable reexpression of Smad4 by retroviral transduction restores TGFβ responsiveness in human SW480 colorectal and BxPC3 pancreatic carcinoma cells

We are using gene transfer techniques to address functions of the tumor suppressor gene Smad4 in human cancer cells. Previously, we have established Smad4-positive derivatives from the colon carcinoma cell line SW480 by stable transfection. This cell model proved that reexpression of Smad4 at subphysiological levels was adequate to suppress tumor growth whereas TGFβ resistance of the cells was retained. Rather, Smad4 induced mesenchymal to epithelial reversion through induction of E-cadherin [15,17]. We also found that Smad4-positive SW480 cells in contrast to Smad4-negative clones deposited an adhesive matrix *in vitro *on tissue culture plastic. Based on this observation we identified the heterotrimeric BM component LM-332 as a novel target structure of Smad4. In pancreatic carcinoma cells BxPC3 and CFPAC-1, Smad4 reexpression also increased constitutive LM-332 expression levels and additionally restored TGFβ induction of LM-332 [19].

**Figure 1:**
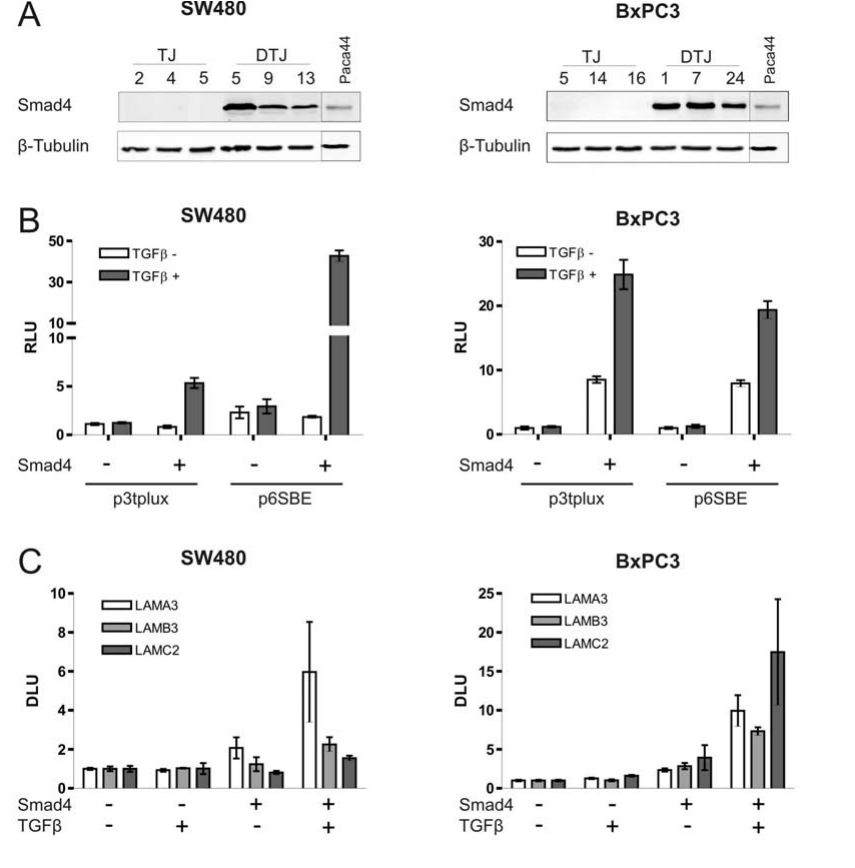
**Restoration of TGFβ responsiveness through reexpression of Smad4**. Smad4 expression was stably restored by retroviral transduction in Smad4-deficient human SW480 colon carcinoma and BxPC3 pancreatic adenocarcinoma cells. **A **Western blot analysis for the human Smad4 protein on total protein extracts of each three Smad4-negative and Smad4-positive clones of SW480 and BxPC3 cells, respectively (TJ: empty vector control clones, DTJ: Smad4- (DPC4) positive clones, Paca44 as a representative cell line to compare for "normal" endogenous expression levels). **B **Transient transfections with p3Tplux and p6SBE reporter vectors. Normalized promoter activity of p3Tplux (a fusion construct of the PAI-1 and collagenase-1 promoters harboring AP1 sites) and p6SBE (a 6fold concatemer of the SBE site) as analyzed in transient transfections of TGFβ-treated (24 h) and -untreated Smad4 negative and Smad4 reexpressing cells. Experiments were repeated in triplicates with the three clones shown in Fig. 1A and bars show the mean values with the standard error of the mean. **C **Reexpression of Smad4 in Smad4-deficient human colon and pancreatic carcinoma cells leads to increased basal and/or TGFβ induced laminin-332 expression. Quantification by phosphor image analysis of Northern blot results with RNAs prepared from TGFβ-treated (24 h) or -untreated Smad4-negative and Smad4-positive SW480 (mean value of each three clones analyzed in two approaches) and BxPC3 (mean value of each two clones in two separate approaches) cells, normalized to GAPDH expression.

In SW480 cells TGFβ responses through the canonical pathway are restricted by very low expression levels of the TGFβ type II receptor; Smad4-positive SW480 cells displayed TGFβ responsiveness of the p3Tplux promoter in cotransfections with a constitutively active TGFβ type I receptor construct, only [17]. As these stably transfected SW480 cell clones express "subphysiological" Smad4 levels (roughly one third of "normal" endogenous levels in Smad4-positive cell lines), we asked if higher Smad4 levels as previously obtained through retroviral gene transfer in Smad4-deficient pancreatic carcinoma cells (i.e. BxPC3) were adequate to overcome the limiting receptor levels in SW480 cells. In fact, a novel set of Smad4-positive retrovirally transduced SW480 derivatives showing moderate overexpression of Smad4 (roughly threefold of "normal" endogenous levels in Smad4-positive Paca44 cells, Fig. 1A) displayed strong TGFβ responsiveness in transient transfection assays with p3Tplux (5–6 fold induction) and p6SBE (40 fold induction) promoter reporter constructs (Fig. 1B).

Smad4-positive BxPC3 cell clones display significantly higher activities of both reporter constructs in the absence and in the presence of exogenous cytokine when compared to Smad4-positive SW480 cells. This may be due to different levels of autocrine TGFβ (family) cytokines expressed by both cell lines. Moreover, we have shown previously in SW480 cells, unlike BxPC3, that very low expression levels of the TGFβ type II receptor restrict TGFβ responsiveness [15].

We then analyzed TGFβ responses of the endogenous genes encoding LM-332 through Northern blot analysis. The results confirmed that moderate overexpression of Smad4 was adequate to restore TGFβ responsiveness of LM-332 in SW480 and BxPC3 cells (Fig. 1C).

### Promoter-reporter constructs of the LAMA3, LAMB3 and LAMC3 genes reflect Smad4-dependent LM-332 induction of endogenous genes

**Figure 2:**
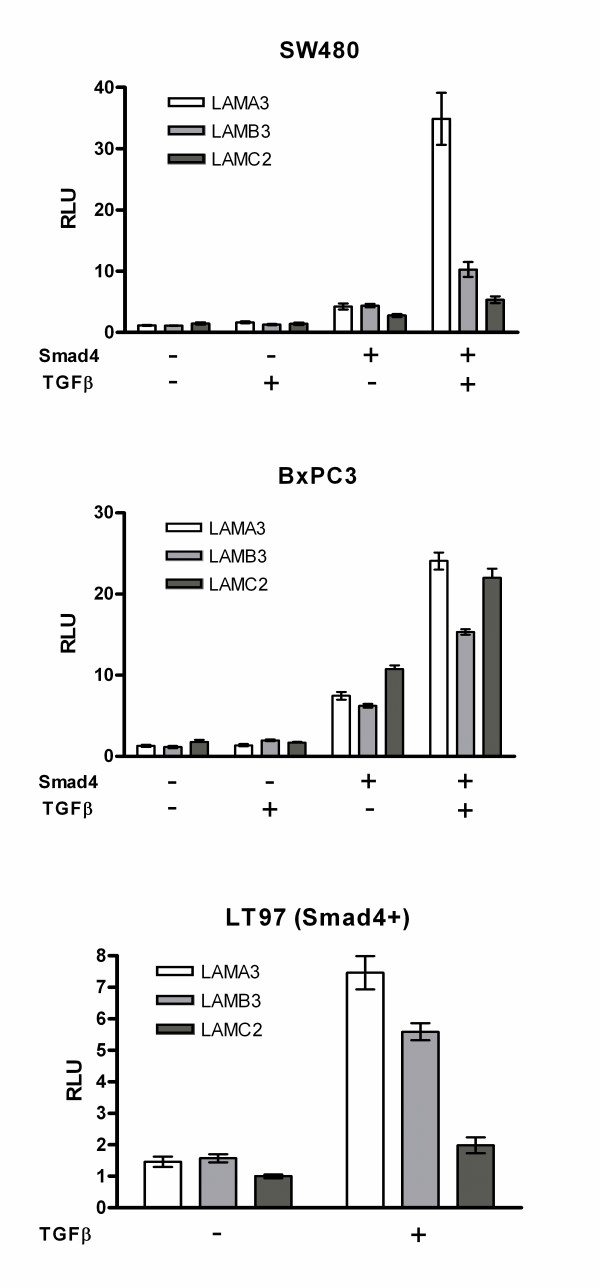
**Transient transfections with 4 kb promoter fragments of the three genes encoding LM-332**. Normalized promoter activity as analyzed in transient transfections of each three Smad4-negative and Smad4-reexpressing SW480 and BxPC3 clones and of Smad4-positive LT97 adenoma cells. Bars show the mean value with the standard error of the mean. All three promoter constructs displayed increased constitutive activities in the Smad4-positive derivatives and all of them mediated Smad4-dependent TGFβ induction.

To unravel molecular mechanisms and pathways involved in Smad4-mediated positive regulation of the LAMA3, LAMB3 and LAMC2 genes, we here set up detailed promoter analyses. An *in silico *sequence analysis using MatInspector software [33,34] confirmed that the previously analyzed SBE element at -1.5 kb is the single SBE site in a 5 kb fragment upstream of the transcription start site of the LAMA3 gene [19] and indicated that both, the LAMB3 and the LAMC2 promoters harbor three putative SBE sites approximately at -1.41; 2.66 and 3.67 and at -1.59; 2.67 and 3.85 kb, respectively. Thus, we amplified 4 kb promoter regions from the three LM-332 promoters and cloned them into the pGL3 basic luciferase promoter vector. Next, the activities of the promoter-reporter constructs were tested in Smad4-negative and Smad4-positive clones of the SW480 and BxPC3 cell lines as well as in the Smad4-positive colorectal adenoma cell line LT97 in the absence and in the presence of TGFβ. All three constructs displayed increased constitutive activities in the Smad4-positive derivatives and all of them mediated Smad4-dependent TGFβ induction (Fig. 2) thus reflecting responses of the endogenous genes (Fig. 1C, [19]).

### Smad4-dependent TGFβ induction of the LAMA3 promoter is mediated via an SBE site at -1.5 kb and via downstream AP1 sites

**Figure 3:**
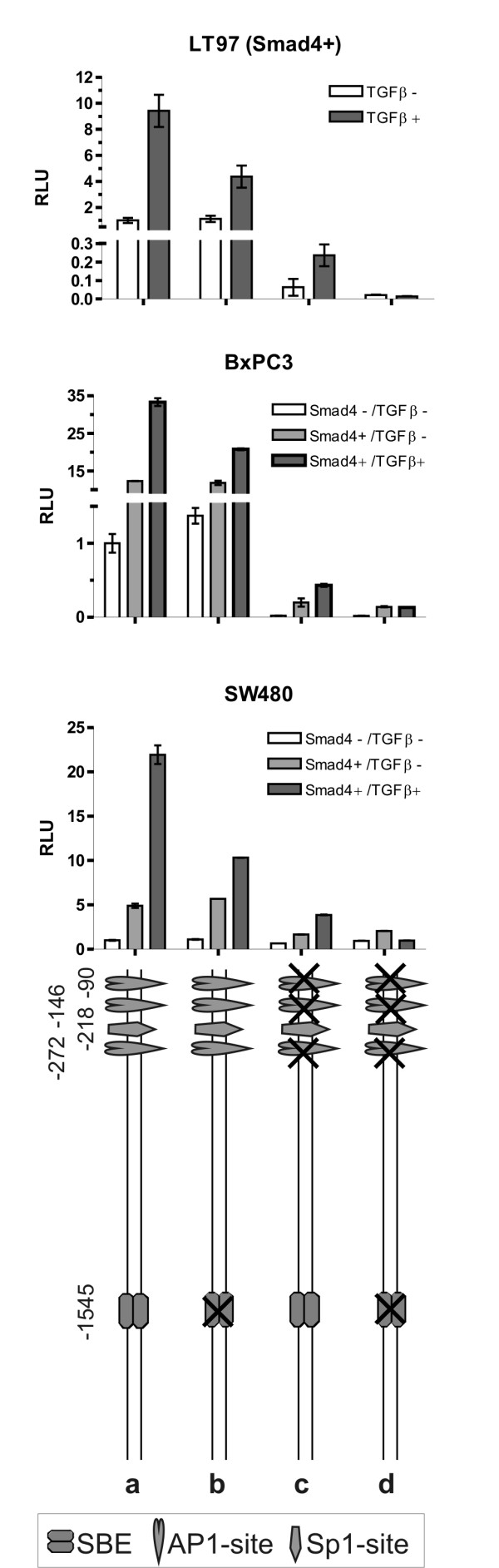
**Smad4-dependent TGFβ induction of the LAMA3 promoter is mediated via an SBE and downstream AP1 sites**. Normalized promoter activity of LAMA3 in Smad4-negative, Smad4-reexpressing and TGFβ-treated Smad4-positive SW480 and BxPC3 carcinoma cells as well as TGFβ-untreated and -treated Smad4-positive LT97 cells. Point mutations were introduced using the Quick-Change Site directed mutagenesis kit (Stratagene). Bars show the mean value of three approaches with the standard error of the mean. Mutational inactivation of the SBE site at -1.5 kb did not affect the Smad4-dependent increase of constitutive activity but reduced the TGFβ response to approximately half in all three cell lines tested (b). A mutation construct with all three AP1 sites mutated displayed a reduction of basal promoter activity (c). The reduction was moderate in SW480 cells and very pronounced in LT97 and BxPC3 cells. When both, mutation of the SBE site and mutation of the AP1 sites, were combined the TGFβ response was completely abolished (d).

The 4 kb (Fig. 2) and the 2 kb LAMA3 promoter constructs displayed very similar responses in all three cell lines analyzed (data not shown). Thus, we used the 2 kb construct for further analyses, which contains the single SBE site (Fig. 3, construct a). Mutational inactivation of the SBE site at -1.5 kb (construct b) did not affect the Smad4-dependent increase of constitutive promoter activity. Moreover, the mutant construct still mediated approximately half of the TGFβ response as compared to the wildtype construct. Thus, we asked for additional promoter sequences involved in Smad4-dependent transcriptional regulation of LAMA3 expression. Three AP1 binding sites close to the transcription start site at positions -90, -146 and -272 in the human promoter confer epithelial specific expression [35] and have also been implicated in the TGFβ response of the mouse promoter [31]. Moreover, AP1 transcription factors are known to interact with Smad proteins [36-38]. A construct with all three AP1 sites mutated displayed a reduction of basal promoter activity as expected (construct c). The reduction was moderate in SW480 cells and very pronounced in LT97 and BxPC3 cells which both display epithelial morphology. Levels of Smad4-dependent constitutive and TGFβ-induced activity were reduced correspondingly. Interestingly, when both, mutation of the SBE site and mutation of the AP1 sites were combined, the TGFβ response was completely abolished (construct d), suggesting that Smad4 cooperates with AP1 transcription factors in TGFβ-induced LAMA3 induction.

### Each of the putative SBE sites in the LAMB3 and LAMC2 promoter is non-functional

**Figure 4:**
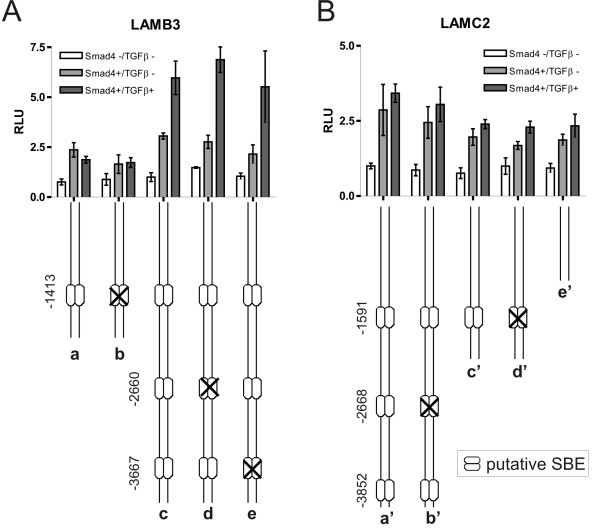
**Putative SBEs in the LAMB3 and LAMC2 promoters prove non-functional**. Normalized and averaged promoter activity obtained from three experiments in Smad4-negative, Smad4-reexpressing and TGFβ treated Smad4-positive SW480 cells. While the -2 kb LAMB3 promoter did not confer TGFβ responsiveness (a) and the mutation construct of the SBE at position -1.41 kb did not alter promoter responses (b), the -4 kb promoter displayed TGFβ induction (c). Mutational inactivation of both of the SBE sites at positions -2.66 and -3.67 kb did not significantly alter reporter responses in transient transfections (d, e). The -4 kb LAMC2 promoter (a') as well as the -2 kb (c') and the -0.8 kb (e') promoter constructs reflected endogenous gene responses; all of them retained TGFβ responsiveness. Correspondingly, mutations of the upstream SBE sites did not significantly affect TGFβ induction (b', d').

To the best of our knowledge functional analyses of the LAMB3 promoter except for methylation studies have not yet been published. As the *in silico *sequence analysis indicated a putative SBE site at position -1413 we first cloned a 2 kb promoter fragment into the reporter vector (Fig 4A, construct a). This construct reflected the Smad4-dependent increase in basal expression levels but did not display TGFβ induction neither in SW480 cells (Fig. 4) nor in BxPC3 cells (data not shown). Moreover, mutation of the putative SBE site at -1.41 kb (construct b) did not alter reporter responses neither in SW480 cells (Fig. 4) nor in BxPC3 cells (data not shown) suggesting that this site is not functional. Next, as two additional putative SBE sites were indicated at positions -2.66 and -3.67 kb, we cloned a reporter construct harboring approximately 4 kb of the LAMB3 promoter (construct c). This construct did show induction in response to TGFβ in all TGFβ responsive cell lines analyzed and thus reflected endogenous gene responses (Fig. 1C, Fig. 2). Surprisingly, however, both of these SBE sites also proved non-functional; as for the SBE site at -1.41 kb mutation of the SBE sites at positions -2.66 and -3.67 kb did not alter reporter responses in transient transfections of SW480 cells (constructs d, e) and other TGFβ responsive cell lines (Smad4-reconstituted BxPC3, LT97, Hacat; data not shown).

The 4 kb promoter construct (Fig 4B, construct a') of the LAMC2 promoter also reflected endogenous gene responses to Smad4, namely Smad4-dependent increase of constitutive expression and a less pronounced Smad4-dependent TGFβ induction of LAMC2 expression (Fig. 1C, 2). The 4 kb region of the LAMC2 promoter harbors three putative SBE sites, located at -1.59; -2.67 and -3.85 kb. Functional regulatory sites were delimited by deletion and mutation constructs (constructs b'-d'). Surprisingly, we found, that a 0.8 kb promoter fragment still showed similar activities in SW480 cells as compared to the 4 kb fragment suggesting that all relevant regulatory sites reside within this part of the promoter (construct e').

### Smad4 effects on constitutive and TGFβ-induced promoter activities of LAMB3 and LAMC2 are conferred through AP1 and Sp1 sites

**Figure 5:**
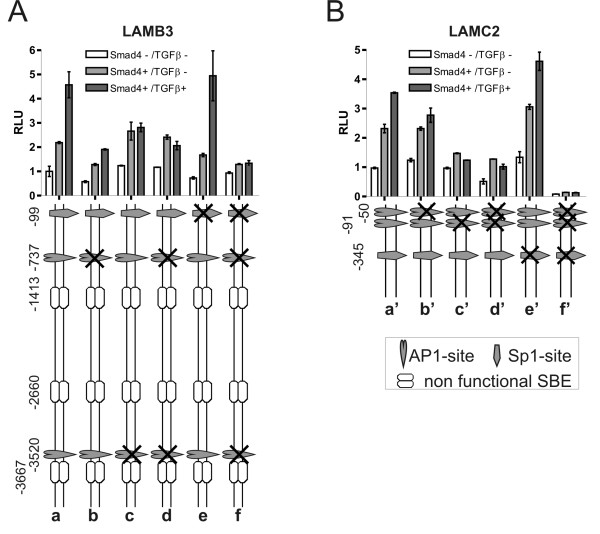
**Smad4 effects on LAMB3 and LAMC2 promoter activities are conferred through AP1 and Sp1 sites**. Normalized and averaged LAMB3 and LAMC2 promoter activity obtained from three experiments in Smad4-negative, Smad4-reexpressing and TGFβ treated Smad4-reexpressing SW480 colon carcinoma cells (a). Mutation of the promoter-proximal AP1 site reduced basal promoter as well as Smad4-dependent constitutive and TGFβ induced LAMB3 promoter activity (b). Mutation of the 3.5 kb AP1 site inactivated TGFβ responsiveness but did not alter constitutive expression levels (c). When both mutations (d) were combined with mutation of the Sp1 site (e) the Smad4 effect on the LAMB3 promoter activity was completely abolished (f). For the LAMC2 promoter a 0.8 kb fragment displayed increased constitutive activities in Smad4-positive cells and mediated Smad4-dependent TGFβ induction (a'). Both AP1 sites are involved in the Smad4 effect on constitutive and TGFβ induced expression levels (b'-d'). When combined with the mutated Sp1 site (e') all promoter activities were significantly suppressed (f').

As all three putative SBE in the LAMB3 promoter did prove non-functional, we then asked if AP1 sites may mediate Smad4 effects on the LAMB3 promoter. AP1 sites reside at positions -737 and -3520; both were mutated separately and in combination. Mutation of the promoter-proximal AP1 site reduced basal promoter activity (in Smad4-negative cells) as well as Smad4-dependent constitutive and TGFβ-induced promoter activity (Fig. 5A, construct b vs. a). Mutation of the 3.5 kb AP1 site in contrast inactivated TGFβ responsiveness but did not alter constitutive expression levels (construct c). Still, when both mutations were combined, the Smad4-dependent difference in basal activity of the LAMB3 promoter was retained (construct d). Ultimately, we tested the Sp1 binding site indicated at position -99 by *in silico *sequence analysis. Mutation of this site on its own had no significant effect (construct e). When combined with the mutated AP1 sites, however, the Smad4 effect on the LAMB3 promoter activity was nearly completely abolished (construct f vs. d).

On the LAMC2 promoter two neighboring AP1 sites at positions -50 and -91 have been reported to mediate synergistic effects of HGF and TGFβ. Of note, these results were obtained in HT29 colon carcinoma cells, which are Smad4-negative [29]. Here we show, that in particular the AP1-site at position -91 is involved in the Smad4 effect on constitutive and on TGFβ-induced expression levels (Fig. 5B, construct a'-c'). As Smad4-dependent differences in the construct with combined AP1 mutations are retained (construct d'), we additionally analyzed the involvement of the Sp1 site located at position -345 in the LAMC2 promoter. Again, mutation of the Sp1 site on its own had no significant effect (construct e'). When combined with the mutated AP1 sites, however, all promoter activities were strongly suppressed (construct f').

In summary, Smad4effects on constitutive and TGFβ-induced LAMB3 and LAMC2 promoter activity in SW480 cells are mediated through AP1 sites and an Sp1 site whereas SBE sites are non-functional in these promoters.

### Chromatin-IP confirms Smad4 binding to AP1 sites in all three promoters but to an SBE site exclusively in the LAMA3 promoter

The comparison of promoter activities in Smad4-negative and Smad4-reconstituted cells does not allow to differentiate between direct effects (Smad4 binds to the respective promoter region) and indirect effects (Smad4 may alter expression patterns of AP1, Sp1 and other transcription factors). To get more insight into the underlying mechanisms, we next we performed transient cotransfections of a Smad4 expression construct with all three wild-type promoter-reporter constructs into Smad4-deficient SW480 cells (data not shown). This led to increased promoter activities to a similar extent as determined in the stable Smad4-positive derivatives suggesting that all three LM-332 genes are direct Smad4 target genes. Thus, we continued to assess direct binding of Smad4 to the respective sites by chromatin IP.

**Figure 6:**
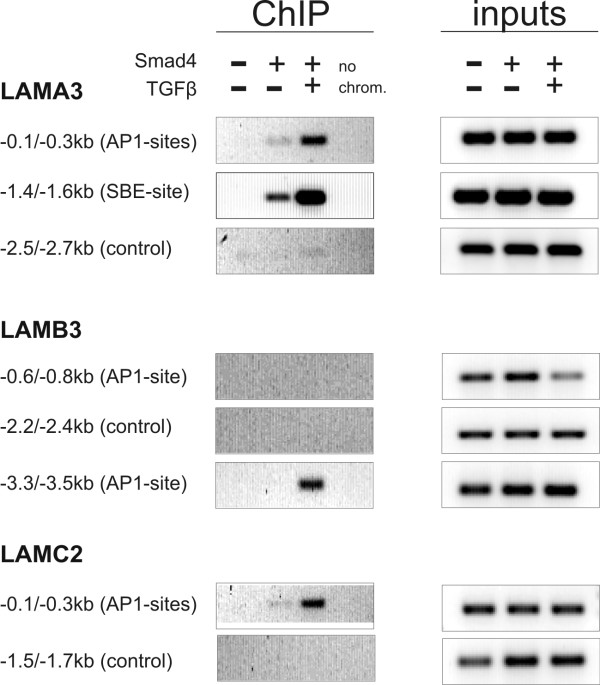
**Chromatin-Immunoprecipitation (ChIP) indicates that Smad4 binds to all three promoters of LM-322**. Chromatin prepared from Smad4-negative, Smad4-reexpressing and TGFβ-treated Smad4-reexpressing BxPC3 cells was shared to obtain fragments of 300–500 bp length, immunoprecipitated and used for PCR amplification of ~200 bp promoter fragments. Input chromatin after sharing is directly used for PCR amplification as a control. Binding of Smad4 to the LAMA3 promoter region which harbors the SBE could repeatedly be shown as well as to the regions which incorporate the functional AP1 sites in all three promoters. Attempts to show binding of Smad4 to other regions within the 4 kb LM-332 promoters failed.

We have shown in our previous work that LAMA3 is a direct target gene of Smad4. Chromatin IP has confirmed Smad4 binding to the SBE region in the endogenous LAMA3 promoter [19]. In this work we demonstrated that the promoter-proximal AP1 sites are additionally involved in conferring TGFβ responsiveness to the LAMA3 promoter in a Smad4-dependent manner (see Fig. 2, 3 construct c). Consistent with this result, Smad4 binding to this region could also be demonstrated by chromatin IP (Fig. 6). PCR amplification of an upstream region performed as a negative control failed.

**Figure 7:**
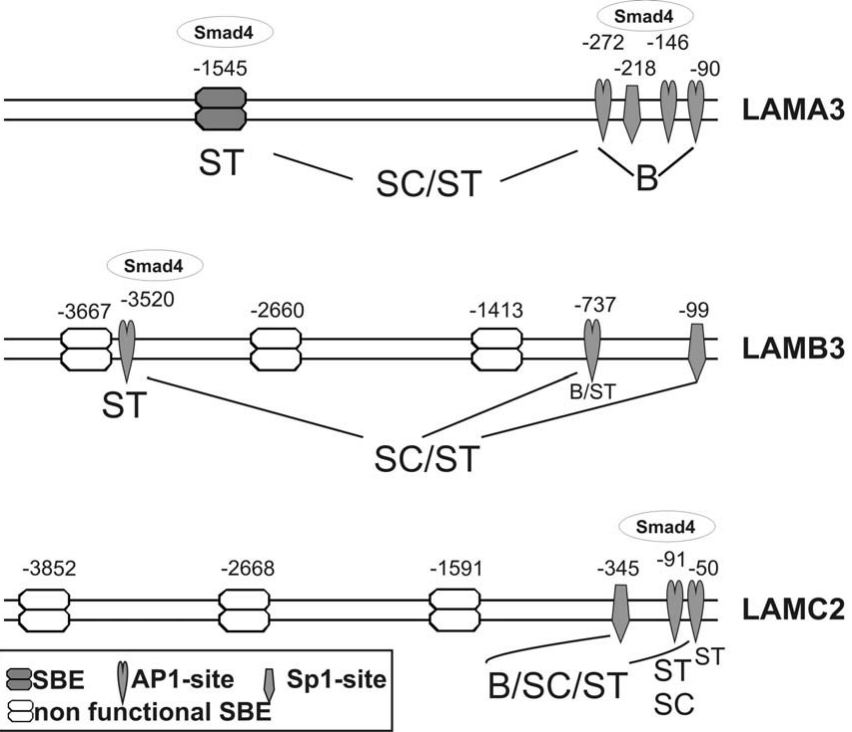
**Compilation of functionally important bindings sites in the three LM-332 promoters**. This scheme shows a summary of results reflecting the complexity of promoter regulation of LM-322. Indicated in the three promoters are the binding sites with significant importance for basal promoter activity "B"; for Smad4 dependent constitutive activity "SC" and for Smad4 dependent TGFβ induction "ST". In addition, binding of Smad4 as confirmed with ChIP analysis is indicated on top of the promoters.

Putative SBE sites in the LAMB3 and LAMC2 promoters, investigated with transient transfections, proved non-functional. Correspondingly, all attempts to show Smad4 binding to the respective promoter regions failed. In contrast, direct binding of Smad4 – presumably in complex with AP1 family transcription factors [36,38] – to the promoter regions with functional AP1 sites could be demonstrated (Fig. 6). In conclusion, results from chromatin IP experiments are consistent with promoter analyses through transient transfections of reporter constructs throughout. The involvement of functional binding sites in basal promoter activity, Smad4-dependent constitutive gene expression and Smad4-mediated TGFβ responses is summarized in Figure 7.

### Smad4 mediates transcriptional induction of AP1 family members in response to TGFβ

**Figure 8:**
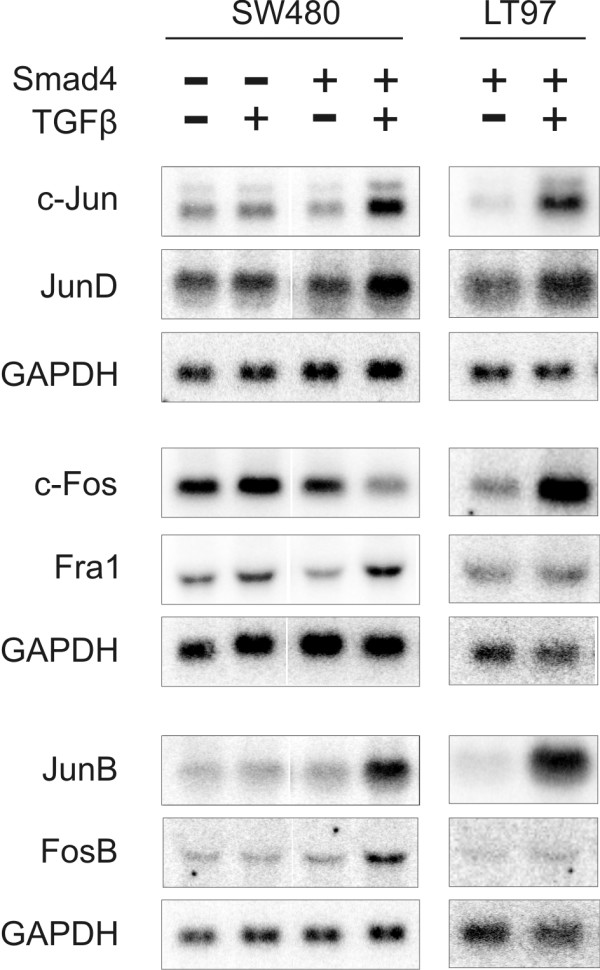
**Smad4 mediates transcriptional induction of AP1 family members in response to TGFβ**. Total RNA from Smad4-deficient and Smad4-reexpressing SW480 cells and Smad4-positive LT97 colorectal adenoma cells was hybridized with specific probes for AP1 family members. Results were confirmed twice with similar results. Expression levels remained unaltered by the addition of recombinant TGFβ in Smad4-deficient SW480 cells. In contrast, all of these genes except for c-fos displayed increased mRNA levels in Smad4-reexpressing SW480 cells in response to TGFβ. Expression of c-fos appeared to be downregulated in Smad4-positive SW480 cells but strongly upregulated in LT97 cells.

Genes encoding AP1 family members have previously been characterized as TGFβ-responsive [39]. Thus, we analyzed transcriptional responses to TGFβ in Smad4-deficient and Smad4-reexpressing SW480 cells as well as in Smad4-positive LT97 colorectal adenoma cells (Fig. 8). Expression levels of c-jun, junB, junD, c-fos, fosB and fra-1 remained unaffected by the addition of recombinant TGFβ in Smad4-deficient SW480 cells (fra-2 not expressed). Smad4-reexpressing SW480 cells, however, displayed increased transcript levels of c-jun, junB, junD, fosB and fralin response to TGFβ as did LT97 cells (with the exception of fosB and fra1). Interestingly, expression of c-fos was downregulated in Smad4-positive SW480 cells but strongly upregulated in LT97 cells. Whereas TGFβ-induced binding of Smads to promoter elements in the c-jun and junB promoters has been reported earlier [37,40] the involvement of Smad4 in transcription regulation of other AP1 family members is a novel finding. Our findings suggest, that "indirect" effects of Smad4, namely altered expression patterns of AP1 family members, may impinge on Smad4 target genes, among them the genes encoding LM-322.

## Discussion

Molecular mechanisms and target genes through which Smad4 mediates its tumor suppressor function are still incompletely understood. We have previously reported that Smad4 is a positive regulator of the three genes which encode for the heterotrimeric Laminin-332 (LM-332) molecule, a prominent component of basement membranes (BMs). Reexpression of Smad4 in Smad4-deficient tumor cells led to secretion and deposition of the heterotrimeric molecule in BM-like structures and was associated with reversion from mesenchymal-like to epithelial morphology, with suppression of invasiveness *in vitro *and suppression of tumor growth *in vivo *[15-19]. Expression control of an essential BM component thus constitutes an important function of the tumor suppressor Smad4.

Smad4 is the central mediator of TGFβ responses through the canonical TGFβ/Smad signal cascade. Smad4 is the single co-Smad that forms complexes with receptor-Smads, which then translocate into the nucleus where they bind to Smad binding elements (SBE) in the promoter region of target genes. In addition, Smad complexes can be targeted to DNA by interacting with ubiquitous sequence-specific DNA-binding transcription factors like AP1 and Sp1 [22]. TGFβ, in addition to the canonical pathway activates further signal cascades like the MAPK/JNK pathways, which in turn can be modified through cross-talk with the Smads [24,25].

Addressing the molecular mechanisms through which Smad4 mediates transcriptional regulation of the three LM-332 genes we here provide evidence for three different mechanisms: First, Smad4 binds to a functional SBE site, this mechanism is operative in the LAMA3 promoter, exclusively. Secondly, Smad4 binds to AP1 (and Sp1) sites in all of the three promoters presumably via interaction with AP1 family components and lastly, Smad4 mediates transcriptional induction of AP1 factors. As AP1 sites are intimately involved in transcriptional regulation of laminin genes this Smad4 impact on AP1 factors may represent an important, though indirect mechanism of how Smad4 could affect target gene transcription even in the absence of direct binding at the respective promoters.

Whereas Smad4 increases constitutive expression levels and TGFβ responses of all three genes encoding LM-332, the underlying mechanisms are surprisingly complex and substantially diverse. To start to decipher mechanisms and pathways involved we here used transient transfection assays using promoter-reporter constructs and showed that their activities strictly reflected responses of the endogenous genes. The colorectal adenoma cell line LT97 used as a model for premalignant cells displayed increased expression of all three LM-332 genes in response to TGFβ. Likewise, SW480 colorectal and BxPC3 pancreatic carcinoma cells showed transcriptional induction of all three genes in a Smad4-dependent manner, whereas their Smad4-negative counterparts remained unaffected by TGFβ treatment. The LAMA3 promoter, only, harbored a functional SBE at -1.5 kb mediating Smad4-dependent TGFβ induction of LAMA3 promoter activity. In addition, downstream AP1 sites were shown to be involved in basal promoter activity and, in combination with the SBE, to mediate Smad4-dependent constitutive and TGFβ-induced promoter activity.

The LAMB3 promoter to our knowledge has not yet previously been characterized. We here report that three putative SBE sites surprisingly proved non-functional through the analysis of mutation and deletion constructs. Rather, mutation of an AP1 site at -737 in the LAMB3 promoter reduced basal promoter activity and mutation of a second AP1 site at -3.5 kb abrogated TGFβ responsiveness. Mutation of a promoter proximal Sp1 site did not show significant effects on its own, but combined mutations of the Sp1 and both AP1 sites fully suppressed Smad4 effects on basal and TGFβ-induced promoter activities. Likewise, Smad4-dependent regulation of the LAMC2 promoter was mediated through two AP1 sites and an Sp1 site residing within a 350 bp downstream promoter region. Of note, whereas Sp1 sites are involved in positive regulation of both the LAMB3 and LAMC2 promoters, the LAMA3 promoter with a downstream Sp1 site mutated displayed strongly increased activity suggesting that in the LAMA3 promoter the Sp1 site exerts transcriptional repression (data not shown).

Chromatin immunoprecipitation showed TGFβ-induced binding of Smad4 to the functional SBE in the LAMA3 promoter and to the promoter regions harboring functional AP1 sites in each of the three promoters, confirming that all three genes encoding LM-332 are direct Smad4 target genes. Binding of Smad4 to AP1 sites may work via interaction of Smads with AP1 transcription factors as previously shown in the regulation of collagenase-I [38], MMP1 [40], interleukin-11 [41] and demonstrated by Liberati et al. [37] and Yamamura et al [42].

In addition, expression analysis of AP1 family transcription factors revealed Smad4-dependent transcriptional induction in response to treatment with TGFβ indicating that direct and indirect mechanisms may converge on these three promoters to regulate LM-332 expression. Of note, whereas TGFβ induced binding of Smads to promoter elements in the c-jun and junB promoters has been reported earlier [37,43] the involvement of Smads in transcriptional regulation of other AP1 family members is a novel finding in this work.

Interestingly, the dual interdependence of Smad4 and AP1 factors is not without precedence. Our data suggest that Smad4, both, impinges on the regulation of AP1 expression as well as depends on AP1 factors for binding to AP1 sites. Likewise, it has been shown earlier that the androgen receptor drives the expression of ETS transcription factors and can then be co-dependent on ETS factors for its recruitment to a subset of promoters [44].

We do not yet know if all of these Smad4 effects are mediated through the canonical TGFβ/Smad signaling pathway. TGFβ can also activate the MAPK pathway among others [24,25]; phosphorylation and activation of AP1 proteins through the MAPkinase pathway has been described long ago [45]. However, all of the TGFβ effects observed in our cell models are dependent on Smad4. So, if other signal cascades in addition to the canonical pathway were involved in transcriptional regulation of the laminin genes in SW480 and BxPC3 cells these also function in a strictly Smad4-dependent manner.

We are only beginning to decipher Smad4 functions in cellular signaling networks. Here, we addressed molecular mechanisms underlying Smad4-dependent regulation of constitutive (cell-autonomous) and of TGFβ-induced transcription of laminin genes. Of note, although Smad4 is a positive regulator of all three LM-332 chains the underlying mechanisms are surprisingly complex and binding sites involved are divergent for the LAMA3 promoter on the one hand and for the LAMB3 and LAMC2 promoters on the other side. We hypothesize that this divergence in modular regulation of the three promoters may lay the ground for uncoupled regulation of LM-332 at the invasive front of tumors where an intracellular accumulation of the γ2-chain can often be observed and represents an impressive molecular marker [46-49]. Invading/budding tumor cells are maximally exposed to cytokines expressed by stromal cells at the invasive front. For example, monocytes/macrophages present in many tumors at high numbers are a major source not only for TGFβ but also for TNFα [50,51]. Interestingly, Korang et al. reported that TNFα in epidermal keratinocytes inhibited LAMA3 but not LAMB3 and LAMC2 transcription [26]. We also observe uncoupled responses of the three LM-332 genes to TNFα in Smad4-deficient but not in Smad4-positive tumor cells (Zboralski et al., in preparation) suggesting that loss of Smad4 may also contribute to uncoupled regulation of LM-332 and consequently to an intracellular accumulation of the γ2-chain. NF-kB binding sites mediating TNFα responses through the canonical pathway are present in all three promoters but have not yet been functionally analyzed. In addition, TNFα can also signal through Sp1 [52] and through AP1 binding sites [45] which we have shown here to be implicated in Smad4-dependent transcriptional regulation of the laminin genes.

## Conclusion

In summary, whereas Smad4 is a positive regulator of basal and of TGFβ-induced promoter activities of the three LM-332 genes, the underlying mechanisms are surprisingly complex and significantly differ between the three promoters. Uncoupled regulation, namely induction of the LAMC2 but not the LAMA3 gene in response to signals derived from the tumor stroma at the invasive front of tumors is an important issue in tumor biology. We here show that multiple transcription factors and binding sites are involved in transcriptional regulation of the LM-332 genes. At least some of them, i.e. components of the AP1 family, are well known to be targeted through cytokines other than TGFβ, cytokines, which may also be present in the microenvironment of invasive tumor cells. Thus, it will be interesting in the future to address the impact of the Smad4 status of tumor cells on transcriptional responses in the context of various environmental stimuli.

## Competing interests

The authors declare that they have no competing interests.

## Authors' contributions

DZ participated in the design of the study, performed plasmid construction experiments and luciferase assays, carried out Northern and ChIP analyses and drafted the manuscript, MB established the novel SW480 and MZ the BxPC3 Smad4 cell system, assisted by SH and AS, SAH sequenced the promoter reporter constructs and WS contributed to the design of the study. IS-W is the PI, designed the study and drafted the manuscript. All authors have read and approved the final manuscript.

## Pre-publication history

The pre-publication history for this paper can be accessed here:



## Supplementary Material

Additional file 1 **Primer sequences**. Primer sequences used for plasmid construction, mutagenesis, ChIP analyses and AP1-probes. Click here for file
