# Supplementary material for: Divergent mechanisms underlie Smad4-mediated positive regulation of the three genes encoding the basement membrane component laminin-332 (laminin-5)
Source: BMC Cancer. 2008 Jul 29;8:215. doi: 10.1186/1471-2407-8-215 (PMC2525660; doi:10.1186/1471-2407-8-215)
Supplement: Additional file 1 — Primer sequences. Primer sequences used for plasmid construction, mutagenesis, ChIP analyses and AP1-probes. [file 1471-2407-8-215-S1.pdf]

**vector construction**		
LAMA3 3' BglII -24	AGTAGATCTGCAGGAACCTCTGCCTCTTC	
LAMA3 5' NheI -2025	GATGCTAGCCGTTTGTGCAAGAAAAGATTG	
LAMA3 5'Kpn-3.8kb	GTACTGGTACCTCTGGAAATGGCACACAATG	Fig. 2, 3
LAMB3 3' -4 XhoI	GATCTCGAGTCTTGAATGTGGGGGTCTC	
LAMB3 3' -1947 Nhe	AGACTGCTAGCTTTATGCAGACCCCCAAGAG	
LAMB3_5'-3887_Kpn	AGTCAGGTACCAAGCTGCATGCACAGTTGTC	Fig. 2, 4, 5
LAMC2_3'-11_Hind	CAGTCAAGCTTCCTTGATCAGGTGGTTTTATCG	
LAMC2 5' -2018 Xho	ACAGTCTCGAGTCACTGTCCCAAAATGATGC	
LAMC2_5'-4034_Xho	ACTGACTCGAGCCACTGATCTGCTTTCTTGC	Fig. 2, 4, 6
**mutagenesis**		
lama3_mut_sbe	CGCACATCTGCCAGTGTTGACAGCACTGTCATCAGC	
LAMA3_MUT_SBE	GCTGATGACAGTGCTGTCAACACTGGCAGATGTGCG	Fig 3b,d
MUT_AP1_A	GACAGCCTTCCTCACCTGGATCCGGCAGGCCCGGGCACTG	
mut_ap1_a	CAGTGCCCGGGCCTGCCGGATCCAGGTGAGGAAGGCTGTC	
MUT_AP1_B	GCGCTCTGGCACAGGCTGGATCCTGTGTGAAGTTTAAAGGTG	
mut_ap1_b	CACCTTTAAACTTCACACAGGATCCAGCCTGTGCCAGAGCGC	
MUT_AP1_C	CGTGTTCCCTGCCCGTGGATCCGCCTGTGATTTAGGGC	
mut_ap1_c	GCCCTAAATCACAGGCGGATCCACGGGCAGGGAACACG	Fig. 3c,d
LAMA3-mSP1_S	GGTGGGTGGAAGGTGATGCATGCATCACCTCACAGGAATTACAG	
LAMA3-mSP1_AS	CTGTAATTCCTGTGAGGTGATGCATGCATCACCTTCCACCCACC	data not shown
lamb3_mAP1_0,7k_S	GTAGCTGCCTGTGAGCTCCTCCCACCTGTG	
LAMB3_mAP1_0,7k_AS	CACAGGTGGGAGGAGCTCACAGGCAGCTAC	Fig. 5b,d,f
lamb3_mut_ap1	GGAAGGGGCACCAGGGAGCTCAAGCTCAGGCC	
LAMB3_MUT_AP1	GGCCTGAGCTTGAGCTCCCTGGTGCCCCTTCC	Fig. 5c,d,f
lamb3_mut_sbe	GATAGTCCAATAAATGTTAGGATCGTGCCCCCTGTAAGCATTCC	
LAMB3_MUT_SBE	GGAATGCTTACAGGGGGCACGATCCTAACATTTATTGGACTATC	
LAMB3-MUT-2.7SBE	CTTTGTAGATATCCAGGGGCTAGCCCGTTCCAGGCAGCAGAAG	
lamb3-mut-2.7sbe	CTTCTGCTGCCTGGAACGGGCTAGCCCCTGGATATCTACAAAG	
LAMB3-MUT-3.7SBE	GAGAAGAGTGTGACCAGTTCTCCGGACAGCTGAGCAGTTGTG	
lamb3-mut-3.7sbe	CACAACTGCTCAGCTGTCCGGAGAACTGGTCACACTCTTCTC	Fig. 4
LAMB3_mSP1_S	GCCAGATTCCTGAGACGAGCTCTGCGGTGGGGCTACAC	
lamb3_mSP1_AS	GTGTAGCCCCACCGCAGAGCTCGTCTCAGGAATCTGGC	Fig. 5e,f
lamc2_mut_ap1b	GAGCCTGTGTTTCAGGGTGGAGCTCACCTGTGAATAAGGAG	
LAMC2_MUT_AP1B	CTCCTTATTCACAGGTGAGCTCCACCCTGAAACACAGGCTC	Fig. 6c,d,f
lamc2_mut_ap1a	GTTTTATCGACTCTTCTACCGAGCTCAGTCCTGACAGGAAGAG	
LAMC2_MUT_AP1A	CTCTTCCTGTCAGGACTGAGCTCGGTAGAAGAGTCGATAAAAC	Fig. 6b,d,f
LAMC2-MUT-1.6SBE	CCAGTTTGCAGCAACCTGTGGTACCCCTACCATTCTTGGGTTC	
lamc2-mut-1.6sbe	GAACCCAAGAATGGTAGGGGTACCACAGGTTGCTGCAAACTGG	
LAMC2-MUT-2.7SBE	GGGGCGTCAAGCCTGAGCTAGCCAGTTGTTTGGATGTGTG	
lamc2-mut-2.7sbe	CACACATCCAAACAACTGGCTAGCTCAGGCTTGACGCCCC	Fig. 4
LAMC2-mSP1_S	GGCCTCCCAGTTTGAGGAAGATGCATGCTGCTGTCTACCTCTGTG	
LAMC2-mSP1_AS	CACAGAGGTAGACAGCAGCATGCATCTTCCTCAAACTGGGAGGCC	Fig. 6e, f
**chromatin-IP**		
LAMA3-CHIP	GTGTAGGGAAACTTCAGACATGC	
lama3-chip	ACTTCCTACCTGACAAGAGTCACC	-1,4/-1.6kb (SBE-site)
LAMA3 A-S	GAGGAAGAGGCAGAGGTTCC	
lama3 a-as	TCCACATAACTCGCTTGCAG	-0.1/-0.3kb (AP1-sites)
LAMA3 E-S	ACCCTTCAGCCCTCTTGG	
lama3 e- as	GACATTTGGTTGCTTCTTGG	-2.5/-2.7kb (control)
LAMB3_CHIP-2.4kb	AAAGGGGTGTGGTACTGTGG	
LAMB3-4kb-seqC	CAGACGTCCCAGGAAAAGAG	-2.2/-2.4kb (control)
LAMB3_CHIP-3.5kb	GCCTGCTTCTCAGTCATTCC	
LAMB3-4kb-seqA	TGGACCAGGTTCTCCTATGC	-3.3/-3.5kb (AP1-site)
bLAMB3-590as	AAAAGGCAAGGTTCATCAGG	
BLAMB3-856S	ATGCTTCAGCCTTTCCTTCC	-0.6/-0.8kb (AP1-site)
CLAMC2-340S	GGCTGCTGTCTACCTCTGTG	
clamc2-69as	CCTGTGTTTCAGGGTGTGAC	-0.1/-0.3kb (AP1-site)
CLAMC2-1683S	GCCTTGGATGAGAAAGTTGG	
clamc2-1457as	TCATGCATGTCATGGAGACC	-1.5/-1.7kb (control)

**AP1-probes**	
cJun_S	ACAGAGCATGACCCTGAACC
cJun_AS	GTGTTCTGGCTGTGCAGTTC
JunD_S	GTGCCCAGGAACTCAGAGAG
JunD_AS	ACACACACACACAACCAACG
JunB_S	CGGCAGCTACTTTTCTGGTC
JunB_AS	TGCTGAGGTTGGTGTAAACG
Fra2_S	TTGTCCAGAAGGGGACAAAG
Fra2_AS	CAGGAGACGCCCTACTCAAG
cFos_S	CCGGGGATAGCCTCTCTTAC
cFos_AS	CCCTTCGGATTCTCCTTTTC
FosB_S	TCTTCCTCCCCTCAACAGTG
FosB_AS	AGAGCCAAAACCATGGTCAG
Fra1_S	CATCGCAAGAGTAGCAGCAG
Fra1_AS	GGCCAGCTCAAGAGAAACAG
